# PET Imaging of CD206 Macrophages in Bleomycin-Induced Lung Injury Mouse Model

**DOI:** 10.3390/pharmaceutics17020253

**Published:** 2025-02-14

**Authors:** Volkan Tekin, Yujun Zhang, Clayton Yates, Jesse Jaynes, Henry Lopez, Charles Garvin, Benjamin M. Larimer, Suzanne E. Lapi

**Affiliations:** 1 Department of Radiology, University of Alabama at Birmingham, Birmingham, AL 35294, USA; vtekin@uabmc.edu (V.T.); yzhang16@uab.edu (Y.Z.); blarimer@uab.edu (B.M.L.); 2 Department of Pathology, Johns Hopkins School of Medicine, Baltimore, MD 21287-0013, USA; cyates@tuskegee.edu; 3 Department of Biology and Center for Cancer Research, Tuskegee University, Tuskegee, AL 36088, USA; jjsqrd@bellsouth.net; 4 Riptide Bioscience, Vallejo, CA 94592, USA; cgarvin@riptidebio.com; 5 MuriGenics, Vallejo, CA 94592, USA; lopezh@murigenics.com

**Keywords:** idiopathic pulmonary fibrosis, bleomycin, macrophages, CD206, PET imaging

## Abstract

**Background/Objectives**: The identification of inflammatory mediators and the involvement of CD206 macrophages in anti-inflammatory responses, along with the synthesis of fibrotic mediators, are crucial for the diagnosis and treatment of Idiopathic Pulmonary Fibrosis (IPF). **Methods**: In this study, the assessment of ^68^Ga-labeled linear and cyclic forms of the RP832c peptide, which demonstrate a specific affinity for CD206 macrophages, was performed to evaluate efficacy for CD206 imaging through PET/CT, biodistribution studies, and CD206 staining in a bleomycin-induced lung injury mouse model (BLM). This model serves as a representative framework for inflammation and fibrosis. **Results**: The findings reveal significant peak PET/CT signals (SUV means), ID/gram values, and CD206 staining scores in lung tissues at one week post bleomycin instillation, likely due to the heightened expression of CD206 in the bleomycin-induced lung injury model. In contrast, the healthy mice exhibited no detectable CD206 staining, lower PET signals, and reduced radiopharmaceutical accumulation in lung tissues at the same timepoint. **Conclusions**: These findings suggest that both linear and cyclic [^68^Ga]Ga-RP832c may function as promising PET imaging agents for CD206 macrophages, and thereby a strategy to non-invasively explore the role of macrophages during fibrogenesis.

## 1. Introduction

Idiopathic Pulmonary Fibrosis (IPF) is a devastating, age-related lung disease of unknown etiologies. The fibrotic response is driven by abnormally activated alveolar epithelial cells where the secretion of excessive amounts of extracellular matrix, mainly collagens, results in scaring and the destruction of the lung architecture [1]. Numerous hypotheses propose that inflammation has a significant impact on IPF and identifying the inflammatory mediators is crucial for the diagnosis and treatment [2,3]. In recent years, the importance of macrophages in the development of IPF has increasingly been recognized. Macrophages, classically activated (M1) or alternatively activated (M2), are involved in the pathogenesis of pulmonary fibrosis. M1 macrophages typically contribute to the inflammatory process, whereas M2 macrophages are involved in anti-inflammatory responses and the production of fibrotic mediators such as TGF-β and PDGF [4]. The macrophage mannose receptor (CD206) is expressed on the surface of M2 macrophages and immature dendritic cells. It contributes to tissue repair, the resolution of inflammation, the induction of immune tolerance, and protection from excessive inflammation [5]. The primary amino acid sequence of the murine macrophage mannose receptor protein reveals a high similarity to that of the human version with an overall identity of 82% at the amino acid level [6]. The non-invasive imaging of CD206 macrophages may assist in elucidating the processes of inflammation and fibrosis, thereby aiding in the development of more effective strategies for IPF. The FDA-approved drug tilmanocept, which is a DTPA-mannosyl-dextran, was recently investigated for targeting CD206 and SPECT imaging of bleomycin-induced lung fibrosis and in ex vivo lung biopsies illustrating the utility of CD206 imaging in fibrosis [7]. To image CD206 macrophages, we employed a CD206 PET imaging probe, which was previously developed by our group using the Gallium-68 labeled linear version of the RP832c peptide [8,9]. In addition, we assessed a second generation where the motivation for synthesizing the cyclic version of the peptide lies in the aim of achieving enhanced stability. The cyclic form of the RP832c peptide was compared to the RP832c linear version in the bleomycin-induced lung injury mouse model (BLM). PET imaging of CD206 macrophages was conducted with Gallium-68 labeled linear and cyclic RP832c in BLM and healthy mice. Biodistribution, as well as the staining of lung tissues utilizing CD206 immunohistochemistry (IHC), Hematoxylin/Eosin (H&E), and Picro Sirius Red (PSR), was used to biologically validate our findings.

## 2. Materials and Methods

### 2.1. Radiolabeling

[^68^Ga]GaCl_3_ (15–20 mCi; 555–740 MBq) was obtained from a commercial ^68^Ge/^68^Ga generator (GalliaPharm, Eckert & Ziegler, Berlin, Germany) via the elution of 10 mL (flowrate of the elution; 1 mL/min) of 0.1 M HCl through the generator to a SCX cartridge (Bond Elut SCX Cartridges, 100 mg, particle size 40 μm, Agilent, Santa Clara, CA, USA). Trapped [^68^Ga]GaCl_3_ was eluted from the cartridge using 0.2 mL of 5M NaCl/HCl solution.

Labeling and quality control protocols for the 1,4,7,10-tetraazacyclododecane-1,4,7,10-tetraacetic acid (DOTA) conjugated RP832c peptides (RWKFGGFKWR) on a commercial solid-phase peptide synthesizer with purities greater than 95% were followed from our previously published report [8]. For both linear and cyclic [^68^Ga]Ga-RP832c, the highest apparent molar activity of 6835 mCi/µmol was accomplished by adding 10 µg of linear or cyclic RP832c (1 mg/mL aqueous solution), 1M sodium acetate buffer (pH 4.5) (1:1, *v*:*v*), and 500 µCi (18.5 MBq) of [^68^Ga]GaCl_3_. For in vivo studies, 10 µg of linear or cyclic RP832c (1 mg/mL aqueous solution) was labeled with 100–150 µCi (3.7–5.55 MBq) of [^68^Ga]GaCl_3_ in 1 M sodium acetate buffer (pH 4.5) (1:1, *v*:*v*). The reaction was incubated at 95 °C for 15 min (300 rpm). The radiolabeling yield was assessed using Thin-Layer Chromatography (TLC) and High-Performance Liquid Chromatography (HPLC). TLC was performed using 50 mM DTPA solution and iTLC (glass microfiber chromatography paper impregnated with a silica gel, Agilent, Santa Clara, CA, USA). HPLC was carried out using a C-18 column (Kinetex, 5 µm EVO C18 100 Å, 150 × 4.6 mm, Phenomenex, Torrance, CA, USA) and a gradient elution system utilized mobile phase A (water containing 0.1% trifluoroacetic acid) and mobile phase B (acetonitrile containing 0.1% trifluoroacetic acid) with a flow rate of 1.0 mL/min. The gradient started with 100% A and transitioned to 100% B over 6.67 min and then reverted to the initial gradient conditions over 4.66 min.

The radiochemical stability of [^68^Ga]Ga-linearRP832c and [^68^Ga]Ga-cyclicRP832c were assessed in phosphate-buffer solution (PBS, pH7) and mouse serum using iTLC with a 50 mM DTPA eluant every hour for 4 h.

### 2.2. CD206 Plate Binding Assay

The affinity of linear and cyclic RP832c to murine CD206 (Recombinant mouse MMR/CD206 protein, R&D Systems, Minneapolis, MN, USA) protein was tested via plate binding study using blocking and non-blocking groups. 48 h before the experiment 1 µg of CD206 (in 0.1 mL 15 mM Na_2_CO_3_, 35 mM NaHCO_3_) was added to each well of 96-well flat bottom plate. The plate was kept at 4 °C for 48 h. On the day of the study, 5 µg (0.1 mL aqueous solution) of linear or cyclic RP832c was added to each well (n = 10) of each blocking group and was incubated at 37 °C for an hour. Then, 2 µg (0.1 µCi) of [^68^Ga]RP832c was added to each well (n = 10 for each group) of both blocking and non-blocking groups. Both groups were incubated at 37 °C for an hour. Wells were rinsed with PBS (pH = 7) three times. Three wells were used as standards. Each well of the blocking and non-blocking groups and the standards were measured via the HIDEX gamma counter (Lablogic, Clair-Mel City, FL, USA). Data were decay corrected, and binding (%) values were calculated according to standards.

### 2.3. PET/CT Imaging and Biodistribution Studies

All animal studies were conducted in compliance with the guidelines for the care and use of research animals established by the University of Alabama at Birmingham’s Institutional Animal Care and Use Committee (IACUC) and approved with the animal protocol number IACUC-22814. 16–20 weeks old male C57BL/6 mice were obtained from Charles River, Wilmington, MA, USA and allowed to acclimatize for at least a week before any procedures were performed. The timeline of animal studies is shown in Figure S3 in Supplementary Materials.

### 2.4. Bleomycin-Induced Lung Injury Mouse Model

For the bleomycin-induced lung injury mouse model, 16–20-week-old male C57BL/6 mice were anesthetized with 2–3% isoflurane in oxygen, and lung injury was performed by intratracheal instillation with a single dose of bleomycin sulfate (USP, cat. No: 1076308) (1.25 U/kg, i.t.) in 100 µL of 0.9% saline (pH 7) [10].

### 2.5. PET/CT Imaging and Biodistribution

One, two, and three weeks post-bleomycin-induced lung injury, mice were anesthetized with 2–3% isoflurane in oxygen and administered linear or cyclic [^68^Ga]Ga-RP832c ((100–150 µCi and 10 µg/per mouse) in trace metal water/saline (1:1, *v*:*v*)) via tail vein. Following the administration of the dose, mice were imaged on a GNEXT small animal PET/CT (Sophie, Springfield, VA, USA) with dynamic 60 min PET acquisition followed by a 3 min CT (80 kVp). Upon the completion of the PET/CT imaging, mice were euthanized under anesthesia, and their organs were collected for biodistribution analysis. One cohort was monitored for a duration of 3 weeks through PET/CT scans to observe the development of pulmonary fibrosis, while healthy mice were utilized as the control group. Organs were weighed and measured using the HIDEX gamma counter. Biodistribution data were analyzed via GraphPad Prism 10. Images were processed and SUVs were calculated using VivoQuant software 2022 (Invicro, Boston, MA, USA).

### 2.6. CD206, Hematoxylin/Eosin (H&E) and Picro Sirius Red (PSR) Staining

Following PET/CT imaging, lungs from all cohorts (bleomycin-induced lung injury model administered with linear or cyclic [^68^Ga]Ga-RP832c, healthy mice administered with linear or cyclic [^68^Ga]Ga-RP832c, and mice not administered bleomycin) were collected and fixed in 10% formalin. After 48 h of fixation, the lungs were transferred to 70% ethanol and after an additional 24 h, samples were sent for preparation of tissue sections. Tissue sections were prepared by the UAB Pathology Core Research Laboratory, Birmingham, AL, USA. Subsequently, tissue sections were stained with CD206 (Mouse MMR/CD206 Antibody (20 µg/mL, Cat: AF2535, R&D, Minneapolis, MN, USA). The secondary antibody was the Rabbit anti-goat IgG (H+L) HRP (1:2000 dilution, Cat no: 81-1620, Invitrogen, Waltham MA, USA). Picro Sirius Red Staining was conducted using a commercial kit (Picro Sirius Red Stain Kit, ab150681, Abcam, Waltham, MA, USA). Collagen was stained in red while muscle fibers and cytoplasm were stained in yellow.

CD206 staining was utilized for the identification of CD206 macrophages. Briefly, slides were placed in each solution (xylene, ethanol, 90% ethanol, and Millipore water, Burlington, MA, USA) for 5 min. Antigen retrieval was performed using citrate buffer (Sigma, C9999, 10×, pH 8, St. Louis, MO, USA). For the primary (CD206 antibody) incubation, 20 µg/mL of CD206 antibody was prepared in solution (0.5 mL PBS, 0.015 g BSA, 0.015 mL Triton X, St. Louis, MO, USA) and samples were incubated at 4 °C overnight. For the secondary antibody incubation, Rabbit anti-goat IgG (H+L) HRP (Cat no: 81-1620, Invitrogen, Waltham, MA, USA) was used with a concentration of 1:2000. After the incubation, slides were placed in Hematoxylin (Epredia™ Signature Series™ 7211, Pittsburgh, PA, USA) for 20 s. Then, the rinsed samples were ready to image.

Picro Sirius Red staining was carried out to detect collagens due to pulmonary fibrosis. The kit is intended for use in the histological visualization of collagen I and III fibers. Picro Sirius Red solution was applied to completely cover the tissue section and then the sample was incubated for 60 min. Slides were rinsed twice in acetic acid solution (0.5%). Slides were rinsed in absolute alcohol and dehydrated in 2 changes in absolute alcohol. The mounting media used was Permount™ Mounting Medium, Pittsburgh, PA, USA. CD206-, PSR-, and H&E-stained samples were imaged using EVOS™ M7000 Imaging System (Invitrogen, Waltham, MA, USA). For IHC semi-quantification, all slides were scored under a light microscope according to the degree of staining (0 points negative for staining, 1 point light yellow, 2 points light brown, and 3 points dark brown) and the range of positivity (1 points 0 to 25%, 2 points 26 to 50%, 3 points 51 to 75%, and 4 points 76 to 100%), and the final scores were summed for analysis.

### 2.7. Statistics

A paired *t*-test was performed on SUV mean data using GraphPad Prism 10 and *p* values were calculated. *P* values of <0.05 were considered significant.

## 3. Results

**Figure 1:**
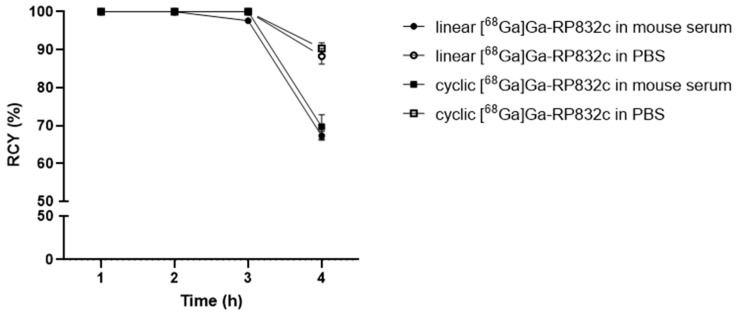
Stability of linear and cyclic [^68^Ga]Ga-RP832c in mouse serum and PBS (pH 7).

Both linear and cyclic [^68^Ga]Ga-RP832c were prepared in radiochemical yields of >99% as assessed with iTLC (Figure S1 in Supplementary Materials) and HPLC (Figure S2 in Supplementary Materials). The % intact for stability of linear [^68^Ga]Ga-RP832c after 4 h was 88.3 ± 2.1 in PBS (pH7) and 67.3 ± 1.2 in mouse serum. The % intact for stability of cyclic [^68^Ga]Ga-RP832c after 4 h was 90.3 ± 1.5 in PBS (pH7) and 69.7 ± 3.2 in mouse serum. Enzymatic activity in the serum environment could potentially contribute to the instability of the peptide observed after four hours in mouse serum. Stability results are shown in Figure 1.

**Figure 2:**
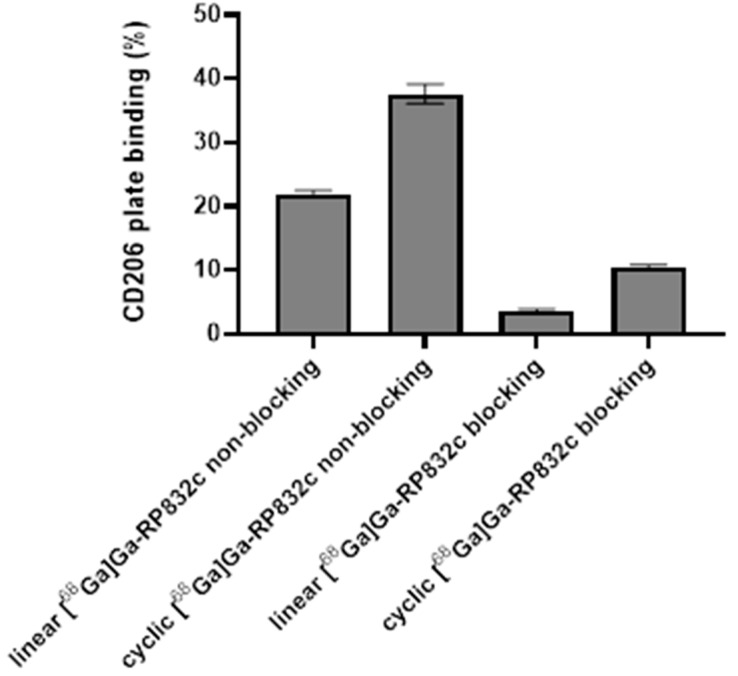
CD206 plate binding (%) of linear and cyclic [^68^Ga]Ga-RP832c (*p* < 0.000001, for both linear and cyclic between blocking and non-blocking).

The CD206 plate binding % of linear [^68^Ga]Ga-RP832c was 21.90 ± 0.52 and 3.51 ± 0.38 for non-blocking and blocking, respectively (*p* < 0.000001). The CD206 plate binding % of cyclic [^68^Ga]Ga-RP832c was 37.60 ± 1.40 and 10.36 ± 0.44 for non-blocking and blocking, respectively (*p* < 0.000001). CD206 plate binding results are shown in Figure 2.

Upon the completion of the radiolabeling and quality control studies, we initiated animal studies employing a longitudinal study. PET/CT images of the longitudinal study are given in Figures S4 and S5. The SUV mean value data of the longitudinal study are given in Figure S6. The observed lung SUV mean values (55–60 min) in the longitudinal study for linear [^68^Ga]Ga-RP832c were 1.01 ± 0.07, 0.78 ± 0.12 and 0.72 ± 0.14 at weeks 1, 2, and 3, respectively. The lung SUV mean values (55–60 min) for cyclic [^68^Ga]Ga-RP832c were 1.17 ± 0.15, 0.65 ± 0.05 and 0.48 ± 0.04, respectively.

**Figure 3:**
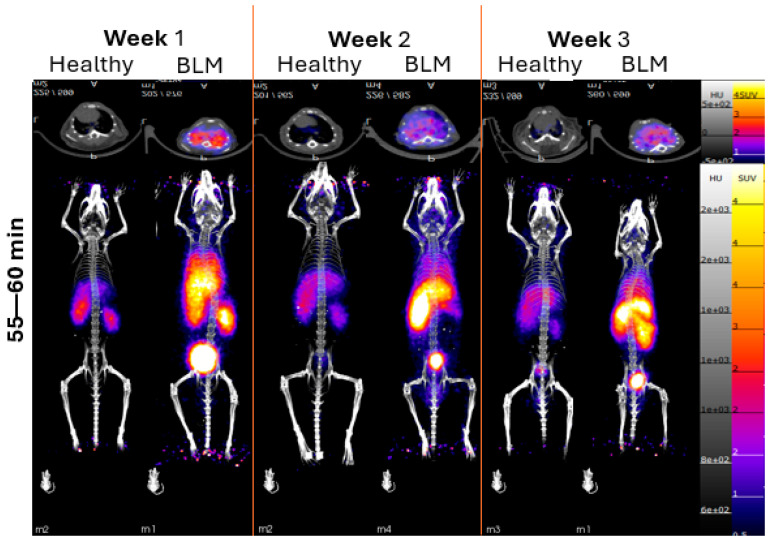
PET/CT images (axial and MIP view, acquired at 55–60 min post-injection time frame) of cyclic [^68^Ga]Ga-RP832c in healthy mice and bleomycin-induced lung injury model. BLM: Bleomycin-induced lung injury model.

**Figure 4:**
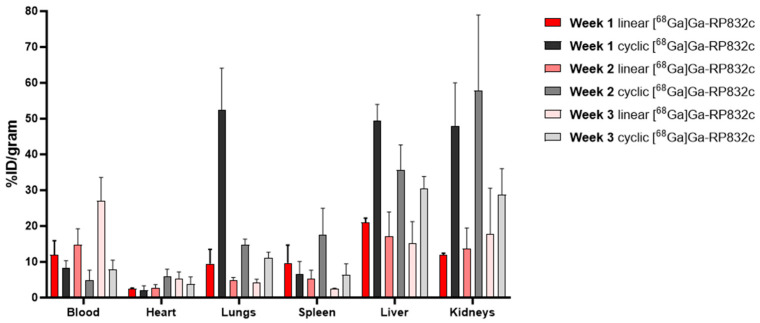
ID/gram (%) comparison between linear and cyclic [^68^Ga]Ga-RP832c at 1-, 2- and 3-week post bleomycin instillation.

The SUV mean value comparisons of both linear and cyclic [^68^Ga]Ga-RP832c in healthy mice and bleomycin-induced lung injury mouse model (BLM) revealed a significantly higher lung uptake in the bleomycin-model (*p* < 0.0001 for each week). The lung SUV mean values (55–60 min) of linear [^68^Ga]Ga-RP832c in BLM and healthy mice were 1.01 ± 0.08 and 0.09 ± 0.01 (week 1), 0.78 ± 0.12 and 0.22 ± 0.04 (week 2) and 0.72 ± 0.14 and 0.20 ± 0.07 (week 3), respectively. The lung SUV mean values (55–60 min) in BLM and healthy mice for cyclic [^68^Ga]Ga-RP832c were 3.71 ± 1.38 and 0.44 ± 0.09 (week 1), 1.53 ± 0.17 and 0.49 ± 0.10 (week 2), 1.69 ± 0.08 and 0.59 ± 0.09 (week 3), respectively. PET/CT images (axial and MIP view, acquired at 55–60 min frame) of cyclic [^68^Ga]Ga-RP832c in healthy mice and in the bleomycin-induced lung injury model are given in Figure 3 and lung SUV mean values are given in Figure S7. The values for linear [^68^Ga]Ga-RP832c are given in Figure S8, and lung SUV mean values are given in Figure S9. The biodistribution following the PET/CT (in Figure 4) imaging showed that the lung %ID/gram values of linear [^68^Ga]Ga-RP832c in BLM and healthy mice were 9.41 ± 4.04 and 5.96 ± 0.62 (week 1), 4.88 ± 0.73 and 4.29 ± 0.86 (week 2), and 4.25 ± 0.86 and 6.70 ± 0.77 (week 3), respectively. The lung %ID/gram values of cyclic [^68^Ga]Ga-RP832c in BLM were found to be significantly higher (*p* < 0.0001) compared to the linear [^68^Ga]Ga-RP832c, which was consistent with the SUV mean values. The lung %ID/gram values were 52.51 ± 11.55 and 7.05 ± 1.65 (week 1), 14.72 ± 1.59 and 5.76 ± 0.37 (week 2), and 11.13 ± 1.50 and 8.26 ± 1.35 (week 3) in BLM and healthy mice, respectively. The trend in lung %ID/gram (approximately 1 h post injection) and SUV mean values (55–60 min) was similar for both the linear and cyclic versions of the peptide. The findings demonstrated that the cyclic version of the peptide behaves similarly to the linear analog with accumulation in the lungs in the BLM model. The total animal and lung weights of healthy mice and bleomycin-induced lung injury model after administration of linear and cyclic [^68^Ga]Ga-RP832c are given in Table S1.

Additional differences were observed between the linear and cyclic versions of the peptide. The liver accumulation at 1 h post-injection of cyclic [^68^Ga]Ga-RP832c in BLM was greater than the linear [^68^Ga]Ga-RP832c in BLM at one week post bleomycin instillation (*p* < 0.01). The liver %ID/gram values of cyclic [^68^Ga]Ga-RP832c were 49.34 ± 4.60, 35.57 ± 7.06 and 30.53 ± 3.30, the liver %ID/gram values of linear [^68^Ga]Ga-RP832c were 18.12 ± 4.16, 32.93 ± 13.24 and 15.08 ± 6.13 at 1-, 2- and 3-week post bleomycin instillation, respectively. The kidney accumulation at 1 h post injection of cyclic [^68^Ga]Ga-RP832c in BLM was significantly greater than the linear [^68^Ga]Ga-RP832c in BLM at 1- and 2-week post bleomycin instillation (*p* < 0.0001. SUV mean (55–60 min) and %ID/gram values of lungs associated with cyclic [^68^Ga]Ga-RP832c in BLM were significantly higher (*p* < 0.0001) compared to those observed with linear [^68^Ga]Ga-RP832c. The higher SUV mean (55–60 min) and %ID/gram values observed in the liver and kidneys for cyclic [^68^Ga]Ga-RP832c (*p* < 0.001) may be attributed in part to the fact that the clearance rate of the linear peptide is more rapid than the cyclic version. The SUV mean (55–60 min) and %ID/gram values of lungs, liver, and kidneys in linear and cyclic [^68^Ga]Ga-RP832c administered healthy mice and bleomycin-induced lung injury model are given in Table S2 in Supplementary Materials.

**Figure 5:**
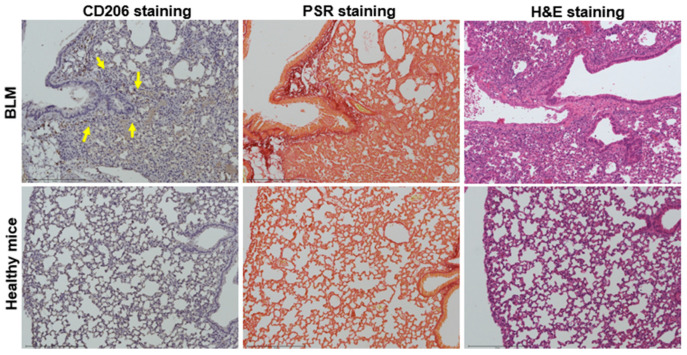
CD206, PSR, and H&E staining in lung samples of healthy mice and bleomycin-induced lung injury model at week 1 timepoint. CD206: Mouse MMR/CD206 Antibody, PSR: Picro Sirius Red, H&E: Hematoxylin and Eosin, BLM: Bleomycin-induced lung injury model. The arrows highlight CD206 staining.

To assess the expression of CD206 in lung slides from the BLM model and healthy animals, IHC staining for CD206 was conducted. Concurrently, the same lung samples underwent staining with PSR to facilitate a visual correlation between the fibrotic tissues and the CD206 IHC staining. Furthermore, H&E staining was utilized to elucidate the anatomical distinctions between the BLM model and healthy control mice. The images of CD206, PSR, and H&E staining in lung samples after cyclic [^68^Ga]Ga-RP832c administration in healthy mice and bleomycin-induced lung injury model at the week 1 timepoint are given in Figure 5. The CD206, PSR, and H&E staining images for the remaining weeks for both linear and cyclic peptides were given in Figures S10–S12 in Supplementary Materials. The findings revealed that CD206 expression was localized in areas corresponding to the fibrotic tissues present in the BLM model. CD206 scores at 1-, 2-, and 3-week post bleomycin instillation were 4.19 ± 0.83, 3.79 ± 0.39 and 3.2 ± 1.55.

**Figure 6:**
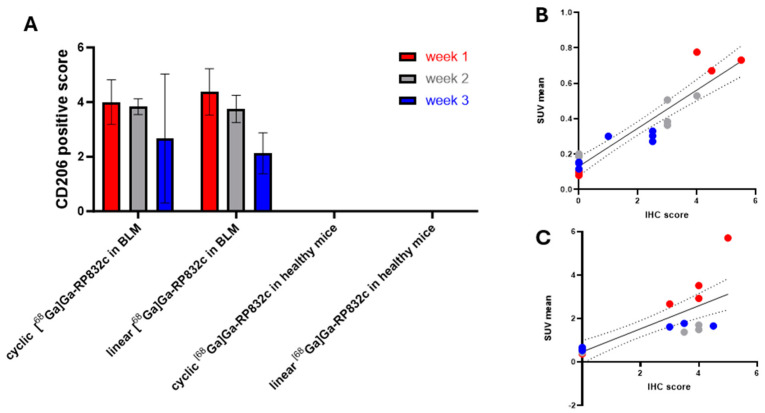
(**A**) The semi-quantification (CD206 positive score) comparison between cyclic and linear [^68^Ga]Ga-RP832c in healthy mice and bleomycin-induced lung injury model at 1-, 2-, and 3-week timepoints (BLM vs. healthy mice in both linear and cyclic [^68^Ga]Ga-RP832c, *p* < 0.0001, 2 way ANOVA, GraphPad, Prism, 10). (**B**) The correlation between lung SUV mean % and CD206 positive score of linear [^68^Ga]Ga-RP832c in healthy mice and bleomycin-induced lung injury model (R^2^ = 0.867, simple linear regression, Prism, GraphPad 10). (**C**) The correlation between lung SUV mean and CD206 positive score of cyclic [^68^Ga]Ga-RP832c in healthy mice and bleomycin-induced lung injury model (R^2^ = 0.637, simple linear regression, Prism, GraphPad 10). BLM: Bleomycin-induced lung injury model.

The cohorts of animals that were administrated with either cyclic or linear [^68^Ga]Ga-RP832c exhibited no significant differences in staining (Figure 6A). The correlation between lung SUV mean % values and CD206 positive score of linear and cyclic [^68^Ga]Ga-RP832c in healthy mice and BLM is given in Figure 6B and Figure 6C, respectively. The quantification of the area % values for PSR staining in BLM measured by ImageJ for cyclic [^68^Ga]Ga-RP832c was 0.86 ± 0.44, 1.28 ± 1.06 and 1.36 ± 0.75 and for linear [^68^Ga]Ga-RP832c was 2.35 ± 0.99, 2.48 ± 0.25 and 1.36 ± 0.44 at 1-, 2-, and 3-week post bleomycin instillation (Figure S13 in Supplementary Materials). No difference in the area % values of the ImageJ quantification for PSR staining in BLM was observed between linear and cyclic [^68^Ga]Ga-RP832c in healthy mice. H&E staining visualized the differences in lung structure between bleomycin-instilled and healthy mice including loss of alveolar gaps and fibrous thickening of alveolar/bronchiolar walls in the bleomycin-treated cohort.

## 4. Discussion

Both linear and cyclic [^68^Ga]Ga-RP832c were prepared in high RCYs (>99%) in agreement with our previous report [8]. Some degradation was observed after 3 h for both linear and cyclic [^68^Ga]Ga-RP832c similar to data reported by Parker et al. for the linear version [8]. The difference between blocking and non-blocking groups for both linear and cyclic versions in CD206 plate binding assay indicated specific binding to CD206 which also aligns with earlier reports with this peptide [9].

CD206+ macrophages play a significant role in the progression of lung fibrosis by exhibiting increased expression levels during the initial stages of the disease [11]. The research conducted by Wang et al. [12] and Zou et al. [13] provides evidence that the expression levels of CD206 in alveolar macrophages (AMs) are consistently elevated in cases of pulmonary fibrosis, as observed in both murine models and patients. Wang et al. reported a notable increase in the population of CD206+ macrophages in the bleomycin-induced mouse model [12], while Zou et al. found that serum concentrations of CD206 were significantly higher in patients diagnosed with idiopathic pulmonary fibrosis [13]. Through a longitudinal study, we investigated the trends in PET signals obtained from CD206 imaging. Our longitudinal study results revealed that the PET signals in the lungs (measured as SUV means) peaked at one week post bleomycin instillation in a 3-week duration in the BLM lung injury model, while the SUV mean decreased two and three weeks after BLM instillation. Subsequent biodistribution studies conducted immediately after PET imaging demonstrated that the lung %ID/gram values were consistent with the SUV mean values, which were highest at one week post bleomycin instillation. A comparison of the lung SUV mean and %ID/gram values between BLM and healthy mice indicated that both linear and cyclic versions accumulated in the lungs in the presence of lung injury. The increased accumulation of both versions in the lungs of the BLM model is likely attributed to the presence of CD206 expression in lung fibrotic tissues. These findings align with the results reported by Pommerolle et al., who observed differences in SPECT signals in ^99m^Tc-tilmanocept imaging between mice receiving either NaCl or BLM [7]. Previous work demonstrates that CD206 expression is significantly upregulated in this model, peaking at one week post bleomycin instillation, which indicates that CD206+ macrophages play an important role during the early phase of lung injury [8]. In the current study, we present both linear and cyclic forms of the RP832c peptide, which exhibit a binding affinity to CD206 as determined by plate-binding assays. These findings may elucidate the relationship between the observed peak PET/CT signals (SUV means) and ID/gram values in lung tissue, alongside the highest levels of CD206 expression noted one week post bleomycin instillation over a three-week period.

H&E staining was used to reveal the anatomical disparities between the BLM model and healthy control mice. The observed differences in the BLM model, such as thickening of the bronchiolar wall and distended blood vessels, decreased alveolar spaces and fibrotic tissues like interstitial fibrosis/collagen were consistent with the findings reported by Ghebremedhin et al., who detailed the distinctions between the control group and bleomycin-induced lung samples [8]. CD206 expression in lung slides from the BLM model was evaluated through the IHC staining of CD206. The results indicated that CD206 expression was specifically observed in regions that aligned with the fibrotic tissues in the BLM model. Additionally, the lung samples were stained with PSR to establish a visual association between the fibrotic tissues and the CD206 IHC staining. The absence of CD206 expression and PSR-stained fibrotic tissues in healthy mice serves as evidence for the existence of CD206 macrophages in BLM, aligning with the findings reported by Pommerolle et al. who demonstrated CD206 and PSR staining outcomes in post-99mTc-tilmanocept imaging among mice received NaCl and BLM [7]. The analysis of CD206 staining in lung tissues from both the BLM model and healthy mice reveals an absence of detectable CD206 staining in the healthy mice, in contrast to elevated scores observed in the lung tissues of the BLM model. Furthermore, a correlation exists between the PET/CT SUV values in the lungs and the CD206 scores from CD206 staining, with higher values recorded in the BLM model and lower values in the healthy mice correlating to imaging with both linear and cyclic [^68^Ga]Ga-RP832c. The results obtained from PET/CT imaging, biodistribution studies, and CD206 staining for both linear and cyclic [^68^Ga]Ga-RP832c consistently demonstrate that the peak values observed at one week post bleomycin instillation are likely attributable to the highest CD206 expression at this timepoint in the bleomycin-induced lung injury model.

## 5. Conclusions

The findings of this study indicate that both linear and cyclic [^68^Ga]Ga-RP832c exhibit a specific binding to CD206 macrophages specifically in the lungs of a bleomycin-induced lung injury model, which serves as a representation of Idiopathic Pulmonary Fibrosis in mice. The demonstrated affinity to CD206 macrophages, along with the differentiation from healthy mice, is substantiated through PET/CT imaging, biodistribution studies, and CD206 staining analyses. This binding affinity holds potential for a PET imaging probe targeting CD206 macrophages. Furthermore, the effective non-invasive PET imaging of CD206 macrophages could advance our comprehension of the underlying mechanisms associated with IPF and facilitate the formulation of diagnostic and therapeutic approaches. In the future. peptide stability could be enhanced through the inclusion of non-natural amino acids. Overall, our study suggests the feasibility of clinical trials imaging CD206 in patients with lung fibrosis.

## Data Availability

The raw data supporting the conclusions of this article will be made available by the authors on request.

## Supplementary Materials

The following supporting information can be downloaded at: https://www.mdpi.com/article/10.3390/pharmaceutics17020253/s1, Figure S1: TLC chromatograms of [^68^Ga]Ga-linearRP832c (a), [^68^Ga]Ga-cyclicRP832c (b) and free ^68^GaCl_3_ (c); Figure S2: HPLC chromatograms of free ^68^GaCl_3_ (a), [^68^Ga]Ga-linearRP832c (b), and [^68^Ga]Ga-cyclicRP832c (c); Figure S3: The timeline of animal studies: longitudinal PET/CT imaging 1-, 2-, and 3-week post bleomycin instillation (a), PET/CT imaging, biodistribution, and tissue collection for staining 1-, 2-, and 3-week post bleomycin instillation (b); Figure S4: PET/CT images (axial and MIP view, acquired at 55–60 min frame) of linear [^68^Ga]Ga-RP832c in longitudinal imaging of 1-, 2-, and 3-week post bleomycin instillation; Figure S5: PET/CT images (axial and MIP view, acquired at 55–60 min frame) of cyclic [^68^Ga]Ga-RP832c in longitudinal imaging of 1-, 2-, and 3-week post bleomycin instillation; Figure S6: lung SUV mean time activity curves of linear and cyclic [^68^Ga]Ga-RP832c in longitudinal imaging of 1-, 2-, and 3-week post bleomycin instillation; Figure S7: lung SUV mean values of cyclic [^68^Ga]Ga-RP832c in BLM: the bleomycin-induced lung injury mouse model and healthy mice at 1-, 2-, and 3-week post bleomycin instillation; Figure S8: PET/CT images (axial and MIP view, acquired at 0–5 min frame) of linear [^68^Ga]Ga-RP832c in healthy mice and the bleomycin-induced lung injury model. BLM: Bleomycin-induced lung injury model; Figure S9: lung SUV mean values of linear [^68^Ga]Ga-RP832c in BLM: the bleomycin-induced lung injury mouse model and healthy mice at 1-, 2-, and 3-week post bleomycin instillation; Figure S10: CD206, PSR, and H&E staining in lung samples of healthy mice and the bleomycin-induced lung injury model at the week 1 timepoint. CD206: Mouse MMR/CD206 Antibody, PSR: Picro Sirius Red, H&E: Hematoxylin and Eosin, BLM: Bleomycin-induced lung injury model; Figure S11: CD206, PSR, and H&E staining in lung samples of healthy mice and the bleomycin-induced lung injury model at the week 2 timepoint. CD206: Mouse MMR/CD206 Antibody, PSR: Picro Sirius Red, H&E: Hematoxylin and Eosin, BLM: Bleomycin-induced lung injury model; Figure S12: CD206, PSR, and H&E staining in lung samples of healthy mice and the bleomycin-induced lung injury model at the week 3 timepoint. CD206: Mouse MMR/CD206 Antibody, PSR: Picro Sirius Red, H&E: Hematoxylin and Eosin, BLM: bleomycin-induced lung injury model; Figure S13: The comparison of PSR staining quantification (ImageJ, area %) between linear and cyclic [^68^Ga]Ga-RP832c in the bleomycin-induced lung injury model at 1-, 2-, and 3-week timepoints. BLM: Bleomycin-induced lung injury model; Table S1: Total animal and lung weights of healthy mice and the bleomycin-induced lung injury model after the administration of linear and cyclic [^68^Ga]Ga-RP832c. BLM: Bleomycin-induced lung injury model; Table S2: SUV mean (55–60 min) and %ID/gram values of the lungs, liver, and kidneys in linear and cyclic [^68^Ga]Ga-RP832c administered healthy mice and the bleomycin-induced lung injury model. BLM: Bleomycin-induced lung injury model.

## Abbreviations

The following abbreviations are used in this manuscript:

IPF	Idiopathic Pulmonary Fibrosis
BLM	Beomycin induced lung injury model
PET/CT	Positron Emission Tomogrophy/Computed Tomography
IHC	Immunohistochemistry
H&E	Hematoxylin/Eosin
PSR	Picro Sirius Red
TLC	Thin-Layer Chromatography
iTLC	Instant Thin-Layer Chromatography
HPLC	High-Performance Liquid Chromatography
DTPA	Diethylenetriamine pentaacetate
MMR	Macrophage mannose receptor
