# Supplementary material for: PET Imaging of CD206 Macrophages in Bleomycin-Induced Lung Injury Mouse Model
Source: Pharmaceutics. 2025 Feb 14;17(2):253. doi: 10.3390/pharmaceutics17020253 (PMC11860134; doi:10.3390/pharmaceutics17020253)
Supplement: Supplementary file 1 [file pharmaceutics-17-00253-s001.zip › pharmaceutics-3434404-supplementary.pdf]

pharmaceutics

MDPI

Supplementary Materials: PET Imaging of CD206 Macrophages in Bleomycin-Induced Lung Injury Mouse Model

Volkan Tekin, Yujun Zhang, Clayton Yates, Jesse Jaynes, Henry Lopez, Charles Garvin, Benjamin M. Larimer and Suzanne E. Lapi

Table S1. Total animal and lung weights of healthy mice and bleomycin induced lung injury model after administration of linear and cyclic [^{68}Ga]Ga-RP832c. BLM: Bleomycin induced lung injury model.

	**linear [^{68}Ga]Ga-RP832c**				**cyclic [^{68}Ga]Ga-RP832c**			
	Healthy mice		BLM		Healthy mice		BLM	
	Animal weight (g)	Lung weight (g)	Animal weight (g)	Lung weight (g)	Animal weight (g)	Lung weight (g)	Animal weight (g)	Lung weight (g)
Week1	26.5 ± 0.7	0.24 ± 0.1	26.8 ± 2.8	0.28 ± 0.1	24.7 ± 2.5	0.16 ± 0.1	22.4 ± 1.1	0.25 ± 0.1
Week2	27.0 ± 4.2	0.15 ± 0.1	27.5 ± 1.3	0.30 ± 0.1	27.3 ± 4.2	0.13 ± 0.1	20.9 ± 0.7	0.23 ± 0.1
Week3	29.5 ± 3.4	0.16 ± 0.1	25.0 ± 3.0	0.37 ± 0.1	29.3 ± 0.5	0.15 ± 0.1	19.5 ± 1.1	0.41 ± 0.1

Table S2. SUV mean (55-60min) and %ID/gram values of lungs, liver, and kidneys in linear and cyclic [^{68}Ga]Ga-RP832c administered healthy mice and bleomycin induced lung injury model. BLM: Bleomycin induced lung injury model.

		**linear [^{68}Ga]Ga-RP832c**				**cyclic [^{68}Ga]Ga-RP832c**			
		Healthy mice		BLM		Healthy mice		BLM	
		SUV mean	%ID/gram	SUV mean	%ID/gram	SUV mean	%ID/gram	SUV mean	%ID/gram
Week1	Lungs	0.09 ± 0.01	5.96 ± 0.62	1.01 ± 0.08	9.41 ± 4.04	0.44 ± 0.09	7.05 ± 1.65	3.71 ± 1.38	52.51 ± 11.55
	Liver	0.85 ± 0.05	18.41 ± 9.20	2.57 ± 1.07	18.12 ± 4.16	0.98 ± 0.06	19.77 ± 2.1	3.74 ± 0.42	49.34 ± 4.60
	Kidneys	0.35 ± 0.04	12.35 ± 5.09	0.74 ± 0.22	11.85 ± 0.54	1.64 ± 0.36	21.46 ± 0.71	4.60 ± 1.93	47.92 ± 12.04
Week2	Lungs	0.22 ± 0.03	4.29 ± 0.86	0.78 ± 0.1	4.88 ± 0.73	1.53 ± 0.17	14.72 ± 1.59	0.49 ± 0.10	5.76 ± 0.37
	Liver	0.84 ± 0.01	11.91 ± 5.13	1.29 ± 0.12	32.93 ± 13.24	1.05 ± 0.12	11.49 ± 2.59	3.62 ± 0.17	35.57 ± 7.06
	Kidneys	0.52 ± 0.20	12.84 ± 5.09	0.87 ± 0.28	13.61 ± 5.80	0.80 ± 0.61	15.65 ± 5.58	6.89 ± 1.22	57.8 ± 21.09
Week3	Lungs	0.19 ± 0.07	4.25 ± 0.86	0.72 ± 0.14	6.70 ± 0.77	1.69 ± 0.08	11.13 ± 1.50	0.59 ± 0.09	8.26 ± 1.35
	Liver	1.59 ± 0.4	18.48 ± 6.17	1.18 ± 0.23	15.08 ± 6.13	3.20 ± 0.63	19.05 ± 3.49	4.32 ± 0.52	30.53 ± 3.30
	Kidneys	0.27 ± 0.07	13.24 ± 10.89	0.74 ± 0.46	16.62 ± 3.20	1.10 ± 0.07	7.39 ± 1.33	3.20 ± 0.96	28.72 ± 7.27

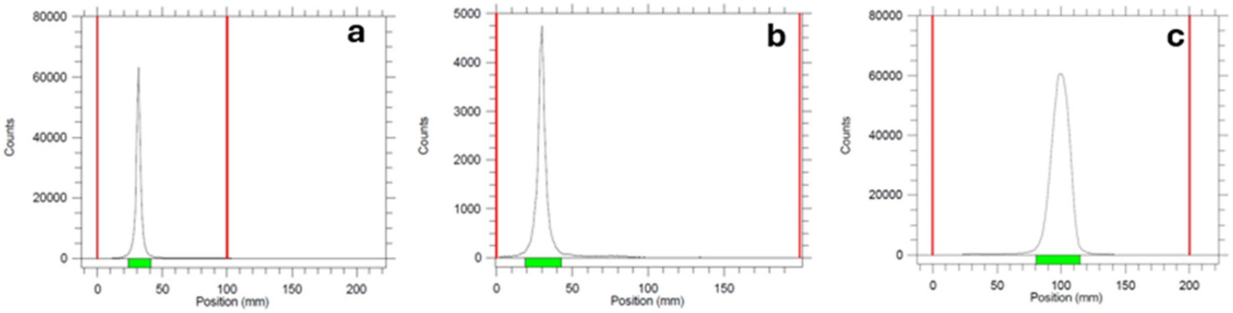

Figure S1. TLC chromatograms of [^{68}Ga]Ga-linearRP832c (**a**), [^{68}Ga]Ga-cyclicRP832c (**b**) and free $^{68}GaCl_3$ (**c**).

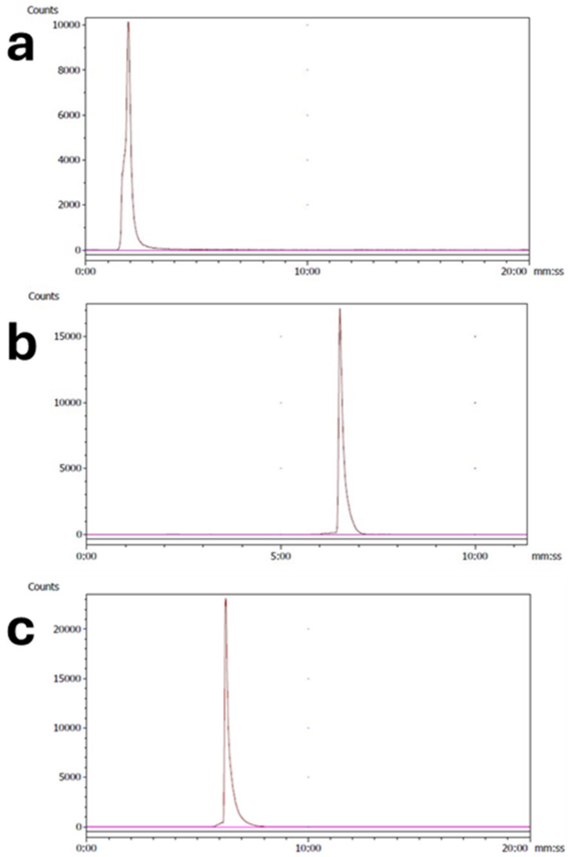

Figure S2. HPLC chromatograms of free $^{68}GaCl_3$ (**a**), [^{68}Ga]Ga-linearRP832c (**b**) and [^{68}Ga]Ga-cyclicRP832c (**c**).

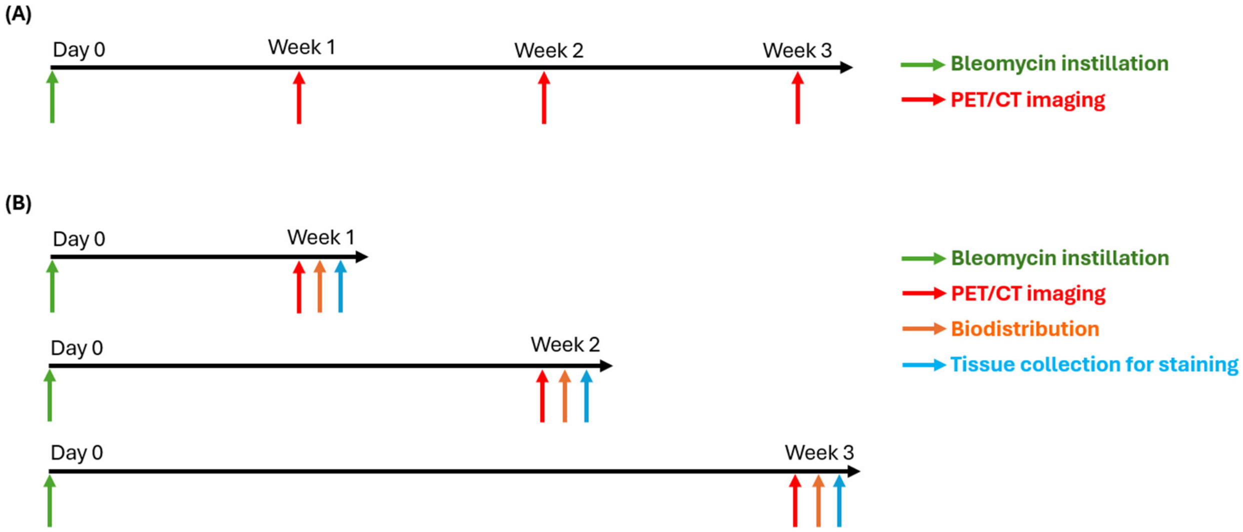

Figure S3. The timeline of animal studies. (**A**) Longitudinal PET/CT imaging 1-, 2- and 3-week post bleomycin instillation. (**B**) PET/CT imaging, biodistribution and tissue collection for staining 1-, 2- and 3-week post bleomycin instillation.

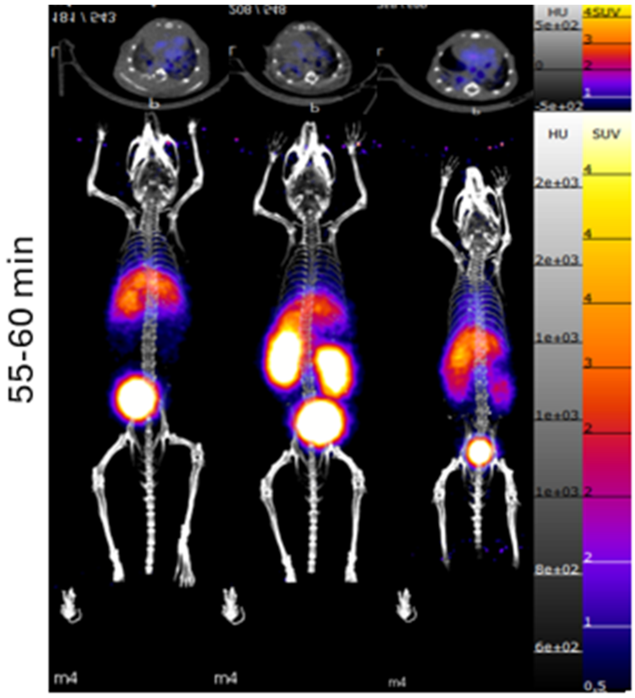

Figure S4. PET/CT images (axial and MIP view, acquired at 55-60min frame) of linear [^{68}Ga]Ga-RP832c in longitudinal imaging of 1-, 2- and 3-week post bleomycin instillation.

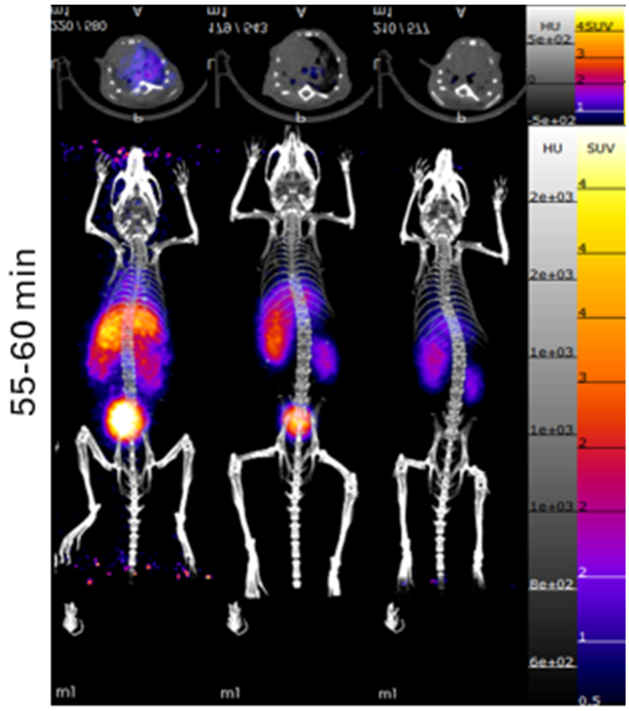

Figure S5. PET/CT images (axial and MIP view, acquired at 55-60min frame) of cyclic [^{68}Ga]Ga-RP832c in longitudinal imaging of 1-, 2- and 3-week post bleomycin instillation.

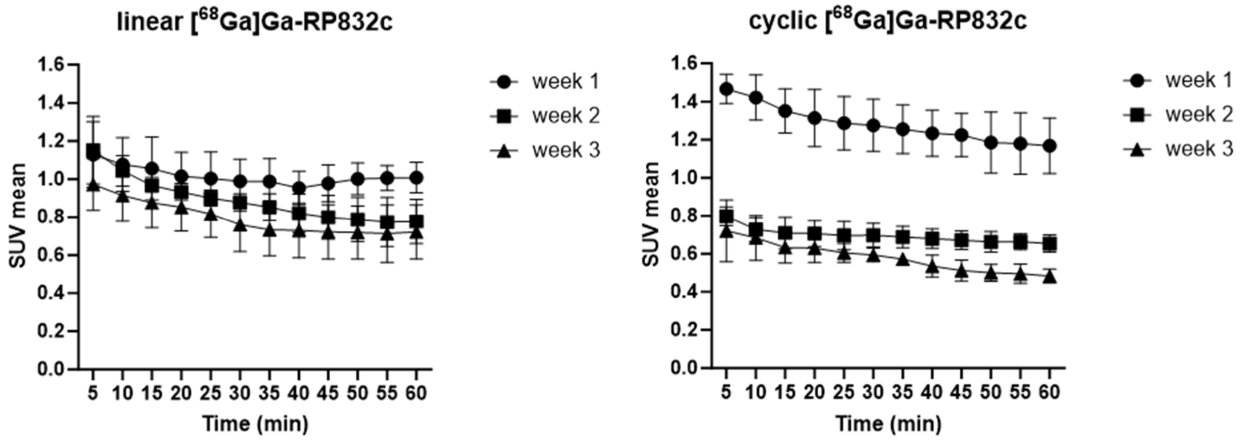

Figure S6. Lung SUV mean time activity curves of linear and cyclic [68Ga]Ga-RP832c in longitudinal imaging of 1-, 2- and 3-week post bleomycin instillation.

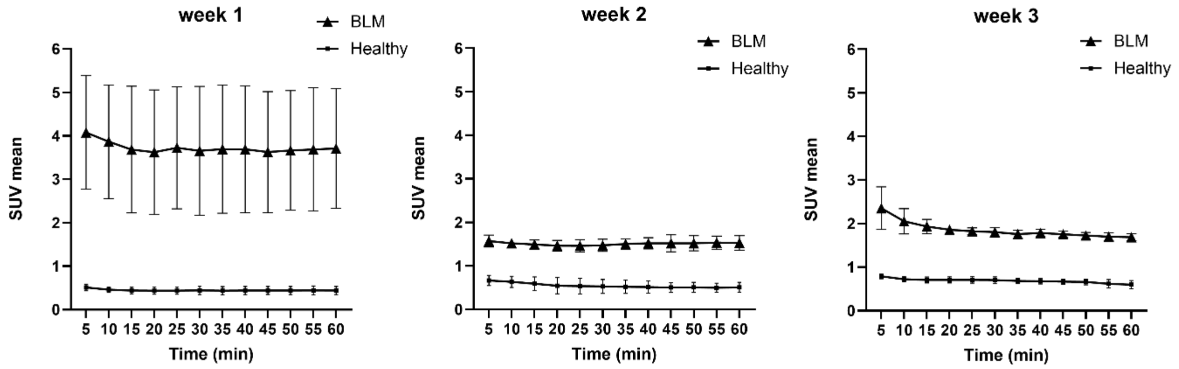

Figure S7. Lung SUV mean values of cyclic [68Ga]Ga-RP832c in BLM: bleomycin induced lung injury mouse model and healthy mice at 1-, 2- and 3-week post bleomycin instillation.

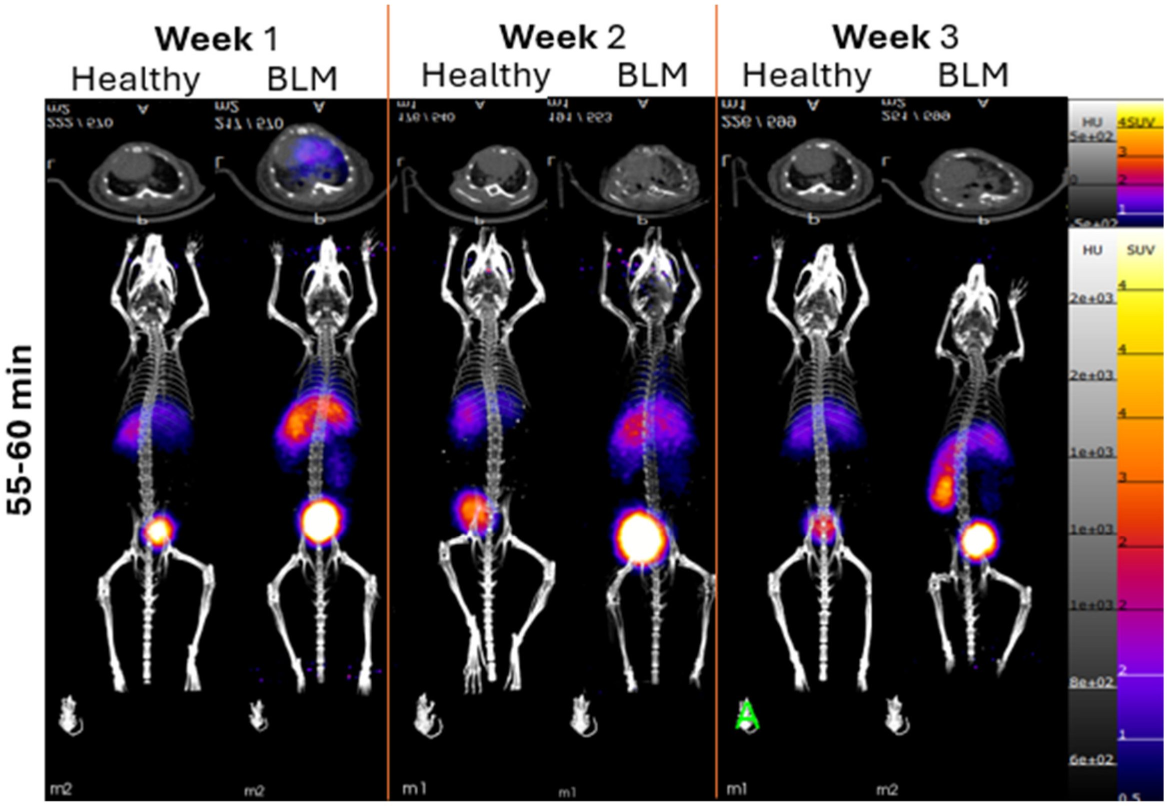

Figure S8. PET/CT images (axial and MIP view, acquired at 0-5min frame) of linear [^{68}Ga]Ga-RP832c in healthy mice and bleomycin induced lung injury model. BLM: bleomycin induced lung injury model.

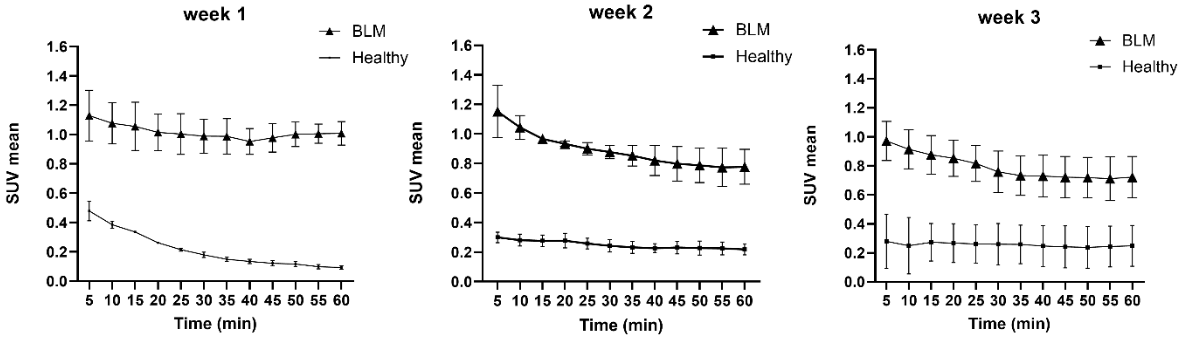

Figure S9. Lung SUV mean values of linear [^{68}Ga]Ga-RP832c in BLM: bleomycin induced lung injury mouse model and healthy mice at 1-, 2- and 3-week post bleomycin instillation.

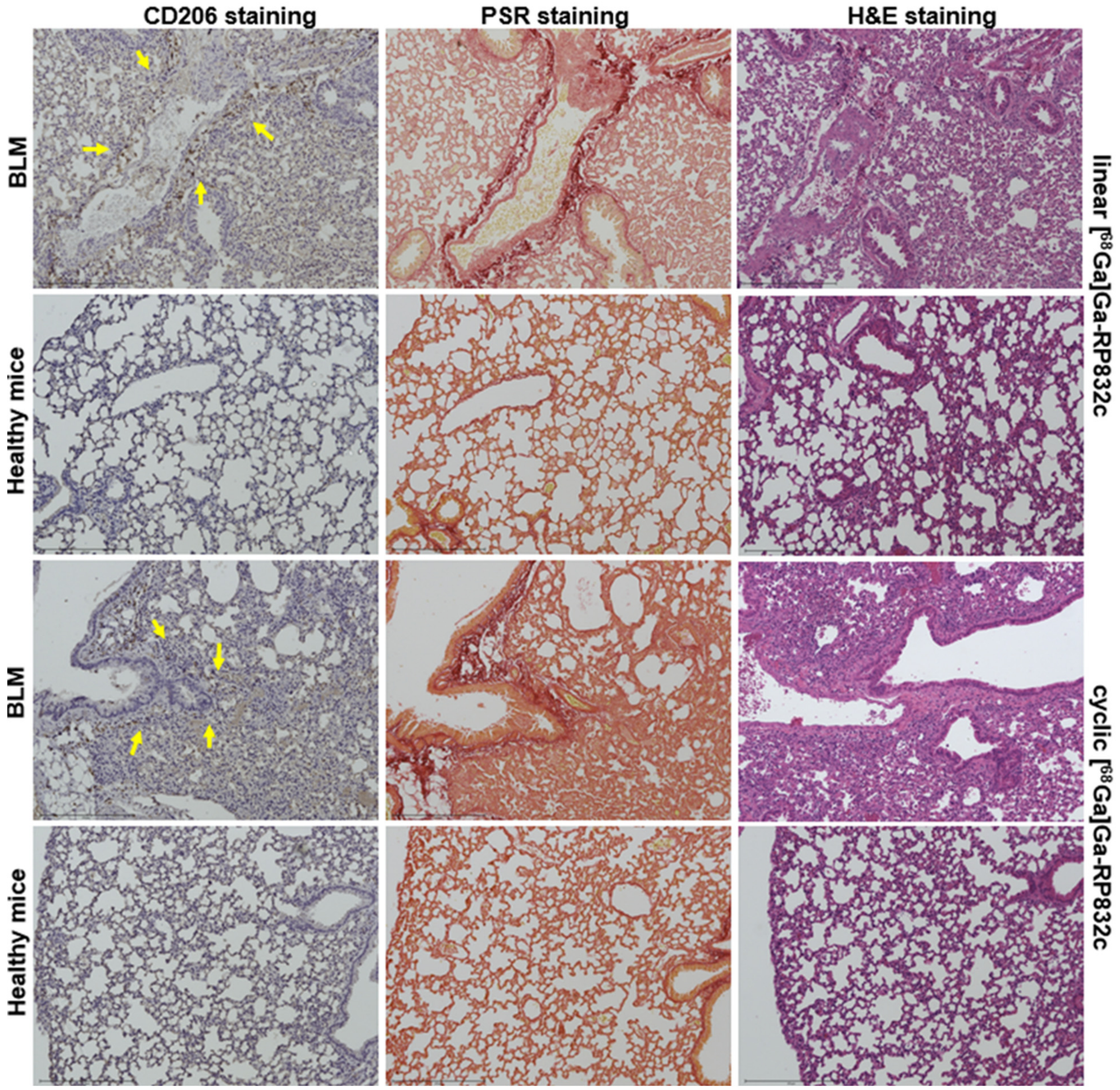

Figure S10. CD206, PSR and H&E staining in lung samples of healthy mice and bleomycin induced lung injury model at week 1 timepoint. CD206: Mouse MMR/CD206 Antibody, PSR: Picro-Sirius Red, H&E: Hematoxylin and eosin, BLM: bleomycin induced lung injury model.

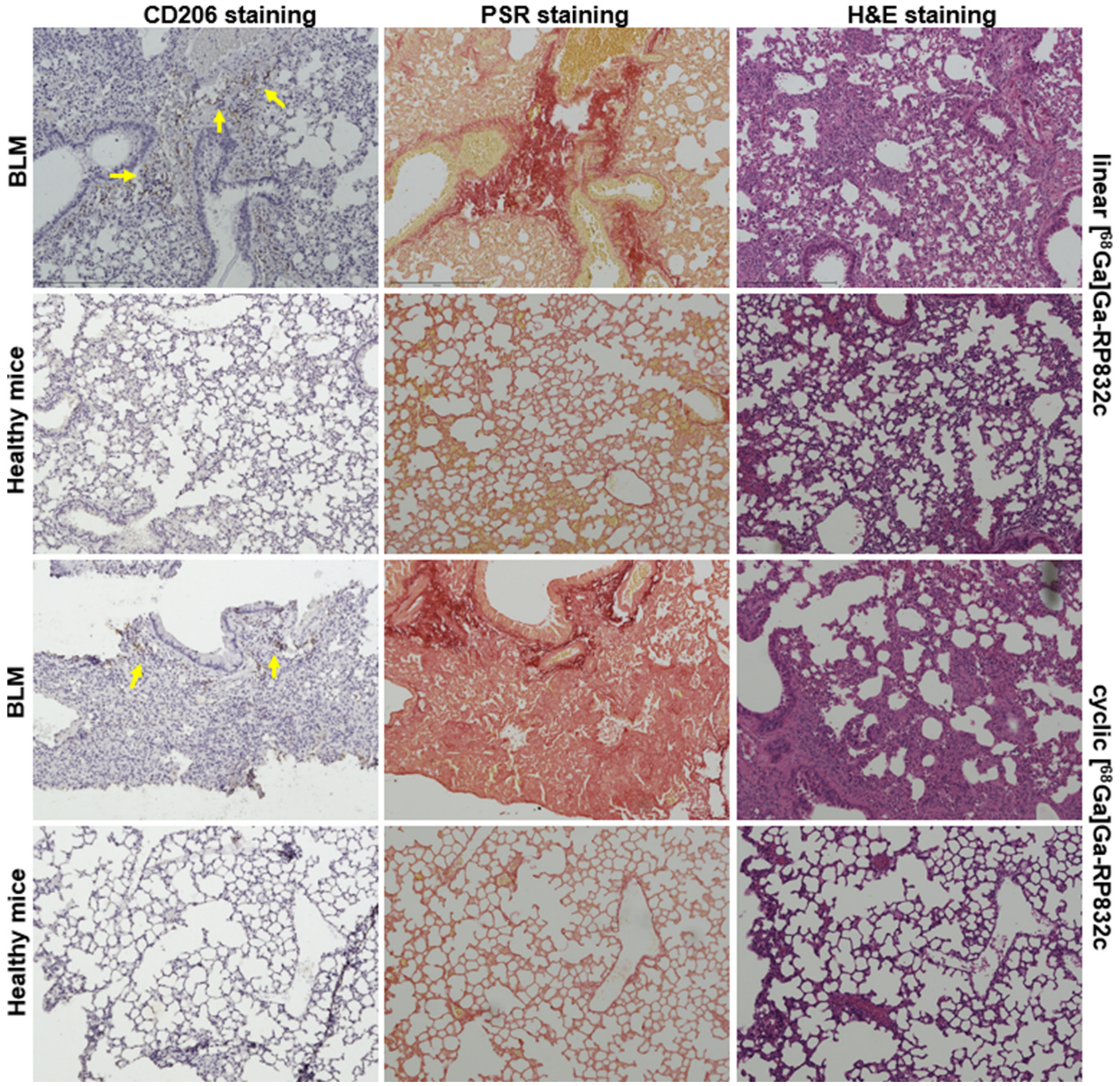

Figure S11. CD206, PSR and H&E staining in lung samples of healthy mice and bleomycin induced lung injury model at week 2 timepoint. CD206: Mouse MMR/CD206 Antibody, PSR: Picro-Sirius Red, H&E: Hematoxylin and eosin, BLM: bleomycin induced lung injury model.

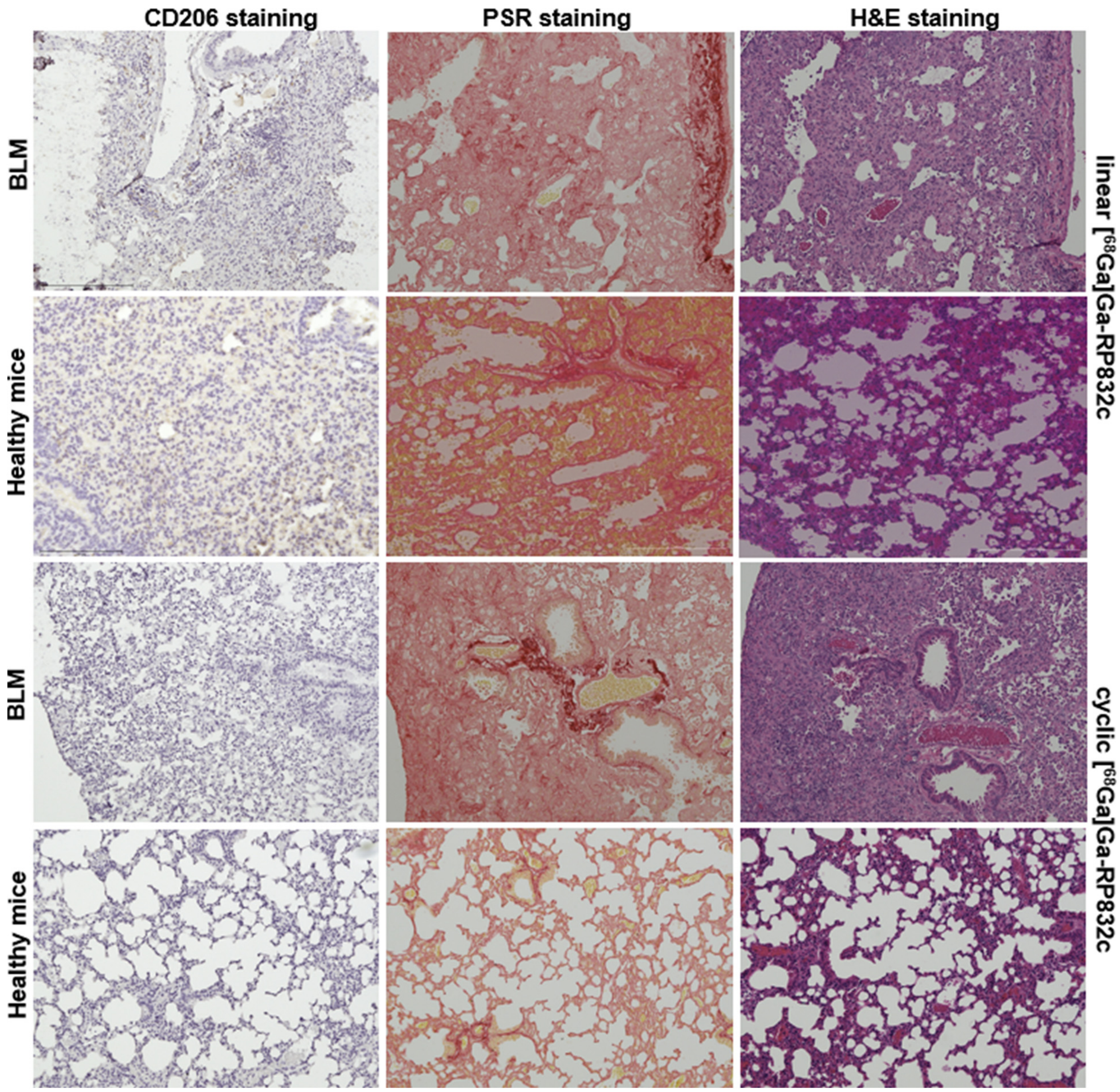

Figure S12. CD206, PSR and H&E staining in lung samples of healthy mice and bleomycin induced lung injury model at week 3 timepoint. CD206: Mouse MMR/CD206 Antibody, PSR: Picro-Sirius Red, H&E: Hematoxylin and eosin, BLM: bleomycin induced lung injury model.

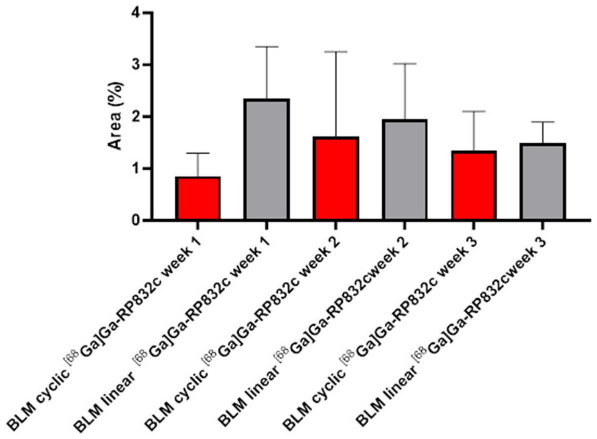

Figure S13. The comparison of PSR staining quantification (ImageJ, area %) between linear and cyclic [^{68}Ga]Ga-RP832c in bleomycin induced lung injury model at 1-, 2- and 3-week timepoints. BLM: bleomycin induced lung injury model.
